# Development and Validation of a Risk Score Predicting Substantial Weight Gain over 5 Years in Middle-Aged European Men and Women

**DOI:** 10.1371/journal.pone.0067429

**Published:** 2013-07-16

**Authors:** Annika Steffen, Thorkild I A. Sørensen, Sven Knüppel, Noemie Travier, María-José Sánchez, José María Huerta, J. Ramón Quirós, Eva Ardanaz, Miren Dorronsoro, Birgit Teucher, Kuanrong Li, H. Bas Bueno-de-Mesquita, Daphne van der A, Amalia Mattiello, Domenico Palli, Rosario Tumino, Vittorio Krogh, Paolo Vineis, Antonia Trichopoulou, Philippos Orfanos, Dimitrios Trichopoulos, Bo Hedblad, Peter Wallström, Kim Overvad, Jytte Halkjær, Anne Tjønneland, Guy Fagherazzi, Laureen Dartois, Francesca Crowe, Kay-Tee Khaw, Nick Wareham, Lefkos Middleton, Anne M. May, Petra H. M. Peeters, Heiner Boeing

**Affiliations:** 1 Department of Epidemiology, German Institute of Human Nutrition Potsdam-Rehbrücke, Nuthetal, Germany; 2 Instiute of Preventive Medicine, Bispebjerg and Frederiksberg Hospitals – Part of Copenhagen University Hospital, The Capital Region, Denmark; 3 Unit of Nutrition, Environment and Cancer, Catalan Institute of Oncology, Barcelona, Spain; 4 Andalusian School of Public Health, Granada, Spain; 5 Department of Epidemiology, Murcia Regional Health Council, Murcia, Spain; 6 CIBER Epidemiología y Salud Pública (CIBERESP), Barcelona, Spain; 7 Public Health Directorate, Asturias, Spain; 8 Navarre Public Health Institute, Pamplona, Spain; 9 Basque Regional Health Department and Ciberesp – Biodonostia, San Sebastian, Spain; 10 Division of Cancer Epidemiology, German Cancer Research Center, Heidelberg, Germany; 11 National Institute for Public Health and the Environment (RIVM), Bilthoven, The Netherlands; 12 Department of Gastroenterology and Hepatology, University Medical Centre, Utrecht, The Netherlands; 13 Dipartimento di Medicina Clinica e Chirurgia, Frederico II University, Naples, Italy; 14 Molecular and Nutritional Epidemiology Unit, Cancer Research and Prevention Institute, Florence, Italy; 15 Cancer Registry and Histopathology Unit, “Civile – M.P.Arezzo” Hospital, Ragusa, Italy; 16 Nutritional Epidemiology Unit, Fondazione IRCCS Istituto Nazionale dei Tumori, Milan, Italy; 17 HuGeF Foundation Torino, Italy; 18 Hellenic Health Foundation, Athens, Greece; 19 WHO Collaborating Center for Food and Nutrition Policies, Department of Hygiene Epidemiology and Medical Statistics, University of Athens Medical School, Athens, Greece; 20 Department of Epidemiology, Harvard School of Public Health, Boston, Massachusetts, United States of America; 21 Bureau of Epidemiologic Research, Academy of Athens, Athens, Greece; 22 Department of Clinical Sciences in Malmö, Clinical Research Centre, Skane University Hospital, Malmö, Sweden; 23 Nutrition Epidemiology Research Group, Department of Clinical Sciences, Lund University, Malmö, Sweden; 24 Section of Epidemiology, Department of Public Health, Aarhus University, Aarhus, Denmark; 25 Danish Cancer Society Research Center, Copenhagen, Denmark; 26 Inserm, Centre for Research in Epidemiology and Population Health, Institut Gustave Roussy, Villejuif, France; 27 Paris South University, Villejuif, France; 28 Cancer Epidemiology Unit, Nuffield Department of Clinical Medicine, University of Oxford, Oxford, United Kingdom; 29 Clinical Gerontology Unit, University of Cambridge, Cambridge, United Kingdom; 30 Medical Research Council (MRC) Epidemiology Unit, Institute of Metabolic Science, University of Cambridge, Cambridge, United Kingdom; 31 School of Public Health, Imperial College, London, United Kingdom; 32 Julius Centre for Health Sciences and Primary Care, University Medical Centre Utrecht, Utrecht, The Netherlands; 33 Department of Epidemiology and Public Health, Imperial College London, London, United Kingdom; Iran University of Medical Sciences, Iran (Republic of Islamic)

## Abstract

**Background:**

Identifying individuals at high risk of excess weight gain may help targeting prevention efforts at those at risk of various metabolic diseases associated with weight gain. Our aim was to develop a risk score to identify these individuals and validate it in an external population.

**Methods:**

We used lifestyle and nutritional data from 53°758 individuals followed for a median of 5.4 years from six centers of the European Prospective Investigation into Cancer and Nutrition (EPIC) to develop a risk score to predict substantial weight gain (SWG) for the next 5 years (derivation sample). Assuming linear weight gain, SWG was defined as gaining ≥10% of baseline weight during follow-up. Proportional hazards models were used to identify significant predictors of SWG separately by EPIC center. Regression coefficients of predictors were pooled using random-effects meta-analysis. Pooled coefficients were used to assign weights to each predictor. The risk score was calculated as a linear combination of the predictors. External validity of the score was evaluated in nine other centers of the EPIC study (validation sample).

**Results:**

Our final model included age, sex, baseline weight, level of education, baseline smoking, sports activity, alcohol use, and intake of six food groups. The model's discriminatory ability measured by the area under a receiver operating characteristic curve was 0.64 (95% CI = 0.63–0.65) in the derivation sample and 0.57 (95% CI  = 0.56–0.58) in the validation sample, with variation between centers. Positive and negative predictive values for the optimal cut-off value of ≥200 points were 9% and 96%, respectively.

**Conclusion:**

The present risk score confidently excluded a large proportion of individuals from being at any appreciable risk to develop SWG within the next 5 years. Future studies, however, may attempt to further refine the positive prediction of the score.

## Introduction

Excess body weight is increasingly recognized as an important public health threat worldwide. In Europe, 30–80% of adults are overweight (Body Mass Index (BMI) ≥25 kg/m^2^) and among them up to 36% are classified as obese (BMI≥30) [1], [2]. Overwhelming evidence suggests that excess body weight is associated with higher risks for numerous chronic diseases [3]. However, not only body weight status per se, but also gain in body weight, irrespective of initial BMI, has been associated with many metabolic abnormalities [4]–[6], subsequently conveying an increased mortality risk [7]. A Danish study further suggests, that weight gain up to the obese level is related to higher risks of impaired glucose tolerance than maintaining weight at the obese level since the beginning of adult live [8].

Given there is no level of safe weight gain, strategies for primary prevention are urgently needed. Even though excess weight is in principle a matter of energy balance, susceptibility to weight gain appears to be determined by a complex interaction between genetic, environmental, socio-economic, cultural and behavioral factors [9]. Much emphasis has traditionally been devoted to the identification of single risk factors that etiologically relate to weight gain or the development of overweight/obesity; however, understanding the combined effects of these risk factors and/or their marker variables is fundamental in order to identify priorities for public health efforts. Additionally, in view of limited resources, prevention efforts may be targeted specifically to those individuals who are at highest risk for gaining substantial amounts of weight and hence associated health risks, and thus – in theory – might benefit most from prevention programs.

In recent years, prediction models to identify high-risk individuals have been proposed for several obesity-related diseases, including cardiovascular disease [10], type 2 diabetes [11], and cancer [12]–[14]. In the present study, we therefore aimed to develop a risk score predicting risk of substantial weight gain (SWG) within the following 5 years among primarily non-obese adults. Because this objective was addressed using data of the multi-center European Prospective Investigation into Cancer and Nutrition (EPIC), the present study additionally offered the unique opportunity to simultaneously investigate the suitability of one universal, trans-european prediction model for SWG.

## Materials and Methods

### Study population

The EPIC study is a multi-center prospective study designed primarily to investigate the relationship between diet, lifestyle and genetic factors and incidence of cancer [15], [16]. Briefly, between 1992 and 2000, a total of 521°330 men and women, aged 25–70 years, were recruited in 23 centers and regions in 10 European countries: Denmark, Sweden, Norway, the United Kingdom, France, Germany, The Netherlands, Spain, Italy and Greece. In the majority of centers, participants were invited from the general population. Exceptions were the French cohort (based on members of the health insurance for teachers), the cohorts in Utrecht (The Netherlands) and Florence (Italy), which are based on women attending local population-based breast cancer screening programmes, components of the Italian and Spanish cohorts (including members of local blood donor associations), and most of the Oxford (UK) cohort (comprising health-conscious subjects, mainly vegetarians). In France, Norway, Utrecht, and Naples only women were recruited. Approval for this study was obtained from the ethical review boards of the International Agency for Research on Cancer (IARC) and from all local institutions where subjects had been recruited for the EPIC study: the Florence Health Authority Ethical Committee (Italy); the Norfolk Local Research Ethics Committee (UK); the Medical Ethics Committee of the Netherlands Organization for Applied Scientific Research (the Netherlands); the Ethics Committee of the Medical Association of the State of Brandenburg (Germany); and the Danish National Committee on Biomedical Research Ethics (Denmark). Written informed consent was obtained from all participants before joining EPIC study.

The prediction model was derived based on data from 6 EPIC centers from 5 countries which participated in the Diet, Obesity and Genes (DiOGenes) project [17], namely: the United Kingdom (UK-Norfolk), the Netherlands [(NL-Doetinchem and NL-Amsterdam/Maastricht); two separate centers because of differences in follow-up assessment of anthropometry], Italy (IT-Florence), Germany (GER-Potsdam), and Denmark (DK-Copenhagen/Aarhus). Subsequently, this model was externally validated in eight remaining EPIC centers.

**Figure 1:**
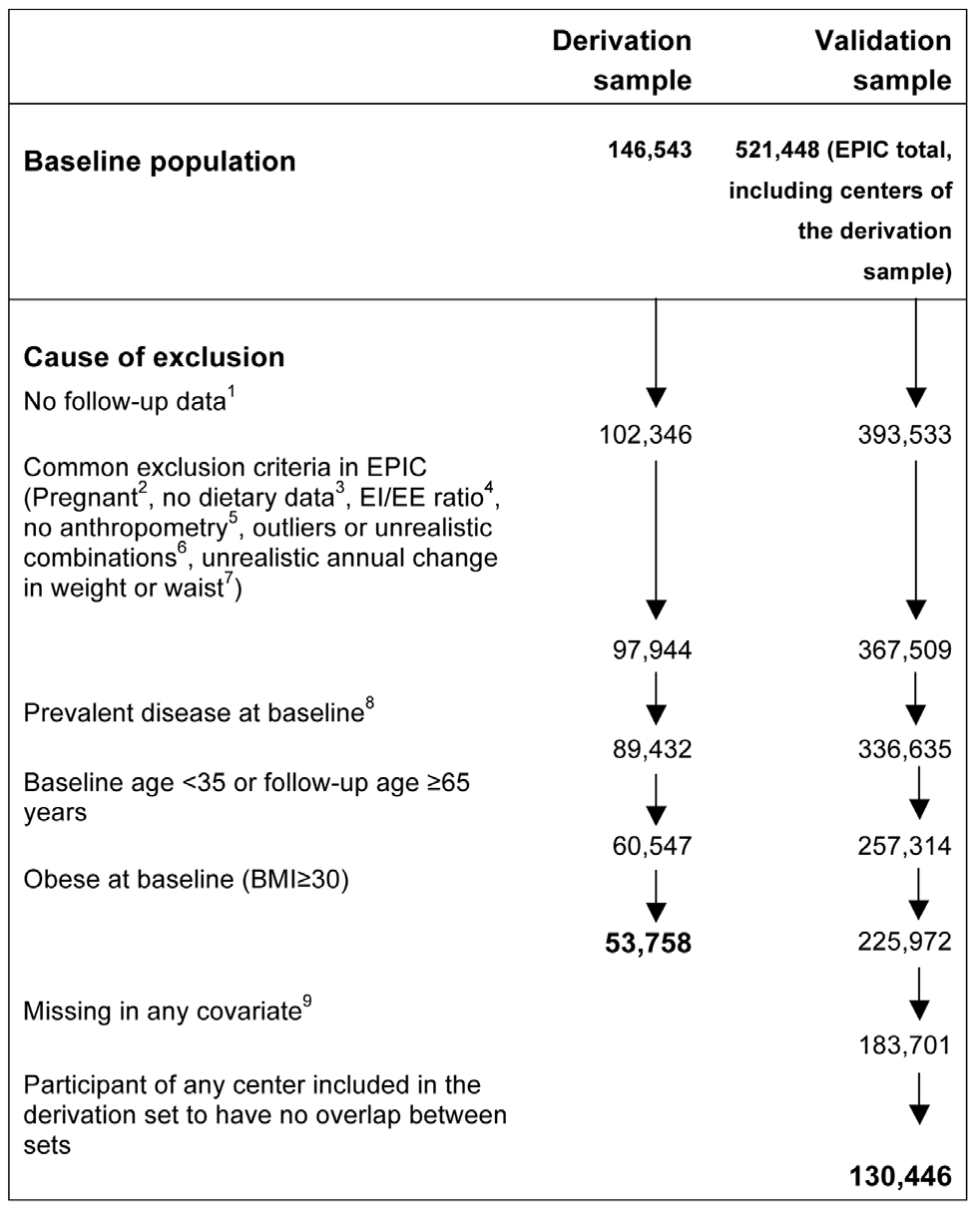
Flow diagram of participants excluded from the present study. ^1^No follow-up questionnaire (e.g. due to death before follow-up body weight assessment, not yet approached for follow-up body weight assessment, emigration or non-response to invitation). ^2^Pregnant at baseline or follow-up. ^3^10% missing items on FFQ. ^4^Ratio of energy intake (EI) to energy expenditure (EE) estimated from predicted resting energy expenditure. ^5^Missing data on baseline or follow-up weight, waist or height, missing follow-up time. ^6^Baseline height<130 cm, BMI<16 kg/m^2^, 0<waist <40 cm, waist>160 cm, follow-up weight>700 kg. Combination of waist<60 cm and BMI>25 kg/m^2^. ^7^Annual weight change>5 kg (either direction) or annual waist change>7 cm (either direction). ^8^ Baseline cancer, diabetes or cardiovascular disease.^9^ In contrast to the derivation of the model where it is important to obtain unbiased estimates of relative risk, we think only original data should be used in the validation sample and we therefore excluded individuals with missing values.

From the 146 543 initial participants in the derivation sample [17], data of 53 758 participants were finally used to guide model development (for flow-chart of exclusions see **Figure 1**). Briefly, exclusions refer to pregnancy and individuals with an extreme ratio between energy intake and energy requirement, to participants who provided no or unrealistic information on anthropometrics at either baseline or follow-up or who reported prevalent CVD, diabetes or cancer at baseline. Additionally, to maintain the same age range in all centers and to minimize confounding from changes in body composition and shape occurring in older age [18] or from undiagnosed chronic disease, the present study was restricted to participants aged ≥35 years at baseline and <65 years at time of the second weight assessment. Finally, the present study was restricted to non-obese individuals (BMI<30) at baseline. After applying the same exclusions and further excluding individuals with missing data in any candidate predictor, the final validation sample consisted of 130 446 men and women, stemming from nine EPIC centers not included in the derivation sample. The centers of Norway and Varese (Italy) were excluded from the validation sample due to missing information on physical activity.

### Dietary and lifestyle assessment

Dietary intake was assessed at baseline by means of validated country-specific dietary questionnaires that were designed to capture local dietary habits and to provide high compliance [16], [19]. Participants were asked to report their average consumption of each food item over the past year. Food intake (gram/day, g/d) was calculated by multiplying the frequency of intake by portion size. The validity and reproducibility of the dietary questionnaires have been shown to be generally good [19], [20]. Information on lifestyle factors was collected by questionnaire and/or face-to-face interview at baseline, including questions on highest level of education, occupational physical activity, sports activity, consumption of alcoholic beverages, and tobacco smoking [16].

### Assessment of anthropometric measures

For each individual, two measures of body weight were available: one at baseline and one at follow-up. In most centers, height and weight were measured at baseline by trained personnel according to standardized procedures [21]. Body weight was corrected to reduce heterogeneity due to protocol differences in clothing worn during measurement by subtracting 1.5 kg in those individuals who were normally dressed and 1 kg in those participants who wore light clothing [21]. In the centers of France and Norway only self-reported anthropometric values were collected. For part of the Oxford center, linear regression models were used to predict sex- and age-specific values from individuals with both measured and self-reported weight (referred to as Oxford prediction equations) [21]. At follow-up, body weight was measured by trained staff in UK-Norfolk and NL-Doetinchem following the same protocol as during baseline measurements, while participants in all other centers measured their weight at home according to guidance provided. The accuracy of these self-reported weights was improved by using the Oxford prediction equations.

### Statistical Approaches

#### Definition of case status

SWG was defined as gaining ≥10% of baseline weight during follow-up. This threshold was chosen for two reasons. First, it was considered major weight gain in relation to the time horizon of the prediction comprising 5 years. Second, it seems high enough to exclude random variation in body weight while simultaneously allowing for some weight gain as natural part of the aging process. Follow-up time, i.e. time between first and second weight assessment, varied considerably between individuals in EPIC (range: 1.2–12.4 years). To best account for these varying follow-up times and to additionally consider the velocity of weight gain, we used methods of survival analysis for statistical analysis. Thus, individual follow-up times (either time to SWG or difference between first and second weight assessment) were used in the proportional hazards model to estimate relative risks, and the results were combined with the baseline survivor function estimated at t = 5 years to provide estimates of 5-year absolute risk as described in detail below. Hence, each participant was followed for incidence of SWG from study entry to the second assessment of body weight (end of follow-up). Those subjects not experiencing SWG during follow-up were censored at time of their second weight assessment and participants experiencing SWG constituted the set of cases. Because it was only possible to determine case status at the time of the second weight assessment, the exact time needed to experience SWG was unknown for the cases. We therefore estimated the time theoretically needed for the cases to cross the threshold of ≥10% baseline-based weight gain by assuming linear weight gain.

### Potential predictor variables

Selection of candidate predictors was primarily based on observed associations with weight change in previous analyses of EPIC and on reported or hypothesized associations in the literature. A total of 21 characteristics were included in the prediction model as candidate predictors. Specifically, we selected standard socio-demographic characteristics, including age, sex, and education, as well as lifestyle factors, namely physical activity (occupational and sports activity), alcohol consumption [22], and smoking status. In view of practical feasibility, selection of dietary factors was restricted to main food items. In accordance with previous EPIC analyses, intake of fruits and vegetables [23], meat [24], bread as indicator of dietary fiber intake [25], complemented by consumption of fish, vegetable oil and dairy products as components of the Mediterranean diet [26], were selected as potential predictors. Additionally, we included intake of butter and margarine, chocolate, cake and cookies, and soft drinks as candidate predictors due to their high energy density and results on health from previous studies [27]–[29].

### Risk prediction model building

Candidate predictors were entered into a proportional hazards model in a stepwise forward model selection process with 0.1 as pre-specified p-values for entering and staying in the model as recommended by Parmer et al. [30]. Interaction terms were not included to keep the model parsimonious and easy to use. To account for heterogeneity between centers due to differences in questionnaire design, follow-up procedures, and other non-measured center effects, stepwise model selection was conducted separately by center. Variables statistically significantly associated with SWG in the same direction in at least two centers and not in the opposite direction were retained as predictors for the final model. Center-specific regression coefficients were obtained for all retained predictor variables by fitting them into a common center-specific model and random-effects meta-analysis was used to calculate combined estimates. Score points (weights) for each predictor were assigned based on the value of the corresponding pooled β-coefficients multiplied by 100 and rounded to two decimal places. For each individual, a risk score was computed as a linear combination of the weighted predictors. The score was rescaled by adding 500 to avoid negative values in descriptive analyses. The probability of experiencing SWG within the following 5 years was finally calculated by inserting the individual risk score points into the survival function obtained from the proportional hazards model. For this, the baseline survival probability at 5 years, i.e. the probability of not developing SWG within 5 years, was estimated separately by center using the average value of each predictor over all individuals in the derivation sample. These center-specific values were again pooled using random-effects meta-analysis.

Because missing data may be associated with bias in estimates of regression coefficients which were used for constructing the risk score, we used multiple imputation techniques in the derivation population [31], [32]. Briefly, in multiple imputation missing data are replaced by several plausible values sampled from their predictive distribution based on the observed data by creating multiple copies of the original data set. Standard statistical methods are being performed in each imputation data set and the results are finally combined by appropriately accounting for the uncertainty about missing data [32]. We used 20 imputation cycles and selection of predictors was performed for each center and separately by imputation data set. As described by Vergouewe et al. [33], predictors that were significantly associated with SWG in at least 50% of the imputed data sets in each center were retained as center-specific predictors from which the final set of predictors was selected as described above.

### Evaluation of the risk score's predictive performance

The predictive performance of the risk score was evaluated by means of discrimination and calibration in the derivation sample (internal validation) and in the independent EPIC centers (external validation). Discrimination was quantified by the c index developed for survival analysis which describes the model's ability to distinguish between persons with longer event-free survival and those with shorter event-free survival within a given time horizon [34], [35]. The c index ranges from a minimum of 0.5 (no discriminatory accuracy) to a theoretical maximum of 1.0 (perfect discrimination).

To define an appropriate cut-off point for the continuous risk score for discrimination between high-risk and low-risk individuals, the Youden's index, a simple measure for which sensitivity and specificity are maximized across a range of possible cut-off values, was used [36], [37]. It is defined as *J  =  sensitivity + specificity –1* and ranges from 0 to 1, with 1 implying perfect separation of diseased and non-diseased by the continuous marker [37].

Calibration, as a measure of how reliable the predictions are, was evaluated by using a modified version of the Hosmer-Lemeshow-Test for survival analysis introduced by D'Agostino and Nam [34]. For this purpose, the observed probabilities of CRC at 5 years estimated by the Kaplan-Meier approach were compared with the average predicted probabilities across tenths of predicted risk which was also plotted for visualization.

Statistical analyses were performed using SAS (Statistical Analysis System, version 9.2; SAS Institute Inc, Cary, NC).

## Results

Among 53 758 men and women in the derivation population, a total of 7°431 individuals gained ≥10% of baseline weight during a median follow-up of 5.4 years, amounting to 329°685 person-years (PY). In the validation sample, 14°622 participants experienced SWG during a median follow-up of 3.7 years (525°749 PY). General characteristics for each center of the derivation sample and the total validation population are presented in **Table 1**. In the derivation population, mean age at baseline was 50.2 years. Mean follow-up time differed considerably between centers, ranging from 3.6 years in UK-Norfolk to 8.8 years in IT-Florence. On average, individuals gained 3.8% of their baseline weight during follow-up, representing a mean annual proportion of baseline-based weight gain of 0.6%. This implies that individuals would need on average 16.7 years to gain 10% of their baseline weight. Due to the all-women centers of France, It-Naples and NL-Utrecht, the proportion of men was substantially lower in the validation sample in comparison to the derivation set (21.5 vs. 41.2%). Mean annual weight gain was higher in the validation than in the derivation sample (521 g/y vs. 395 g/y) which may be explained by the shorter duration of follow-up in the validation sample and by the fact that weight fluctuations are higher over shorter periods of time.

**Table 1:** General characteristics of the derivation and validation population.

	Derivation population														Validation population					
	All	UK-Norfolk	NL-Doetinchem		NL-Amsterdam/Maastricht					IT-Florence	GER-Potsdam	DK-Copenhagen/Aarhus								
N	53,758	6,930	2,951		3,790					5,606	9,859	24,622		130,446						
Men (%)	41.2	42.4	48.4		43.8					22.5	35.3	46.2		21.5						
Age at baseline (y)	50.2 (6.0)	51.9 (5.2)	46.3 (6.8)		45.1 (5.7)					47.4 (5.7)	45.1 (6.3)	53.7 (2.6)		49.1 (6.4)						
Duration of follow-up (y)	6.1 (2.1)	3.6 (0.8)	4.9 (0.4)		9.0 (2.1)					8.8 (1.8)	8.0 (1.5)	5.2 (0.5)		4.0 (2.0)						
**Anthropometry**																				
Weight																				
At baseline (kg)	70.6 (11.5)	69.5 (11.0)	73.7 (11.4)		70.7 (11.4)			64.5 (10.3)			69.3 (11.5)	72.4 (11.4)		65.3 (10.5)						
At follow-up (kg)	73.1 (12.1)	71.2 (11.6)	75.9 (12.1)		75.0 (12.3)			68.2 (11.4)			73.1 (12.3)	74.2 (12.0)		67.5 (11.2)						
Absolute change (kg)	2.6 (4.5)	1.7 (3.6)	2.1 (4.1)		4.2 (5.5)			3.6 (4.9)			3.9 (5.0)	1.8 (4.1)		2.2 (3.6)						
Annual change (g/y)	395 (749)	477 (1022)	429 (823)		433 (569)			384 (517)0			449 (585)	342 (772)		521 (1030)						
Change (% of bl. weight)	3.8 (6.6)	2.5 (5.2)	3.0 (5.6)		6.2 (8.0)			5.8 (7.6)			5.8 (7.4)	2.6 (5.7)		3.4 (5.9)						
BMI																				
At baseline (kg/m^2^)	24.5 (2.7)	24.5 (2.6)	24.7 (2.6)	24.1 (2.7)					24.0 (2.7)		24.4 (2.8)	24.7 (2.7)		24.5 (2.9)						
At follow-up (kg/m^2^)	25.4 (3.0)	25.1 (2.9)	25.4 (2.9)	25.5 (3.2)					25.4 (3.2)		25.8 (3.2)	25.3 (2.9)		24.9 (3.2)						
Obese at follow-up (%)	5.1	6.2	7.7	6.3					6.2		6.8	3.4		5.9						
**Physical activity**																				
At Work (%)																				
Sedentary	43.4	28.8	30.9	38.4			46.1				57.9	43.3		32.5						
Standing	21.6	23.3	21.1	19.7			18.5				28.1	19.5		36.6						
Manual	19.2	22.6	18.1	15.8			9.3				5.1	26.7		7.6						
Non-workers	15.9	25.3	29.8	26.1			26.1				8.9	10.5		23.3						
Sports (hours/week)	1.4 (2.2)	1.7 (3.0)	1.7 (2.5)	1.8 (2.9)			1.3 (2.1)				1.1 (1.8)	1.4 (2.1)		1.4 (2.2)						
**Education (%)**																				
No school/primary school	21.6	26.7	6.8	8.5			35.3				9.3	25.7		24.8						
Techn./profess. school	38.2	46.4	44.7	31.1			12.9				40.1	41.1		15.1						
Secondary school	14.1	10.6	24.8	24.5			29.2				7.2	11.5		28.2						
University degree	26.2	16.3	23.8	36.0			22.6				43.5	21.7		31.9						
**Smoking habits (%)**																				
Never smoker	41.7	51.6	31.5	29.3			40.9				50.1	38.9		55.9						
Former smoker	30.7	34.7	37.6	34.3			29.5				31.1	28.3		24.4						
Current smoker	27.6	13.7	30.9	36.4			29.5				18.8	32.8		19.7						
Alcohol use (%)																				
No alcohol	5.2	15.4	9.7	7.4			10.2				2.1	1.6	14.6							
>0– ≤6g/d	28.7	39.0	35.4	33.4			38.6				37.1	18.6	34.1							
>6– ≤18g/d	33.8	32.4	29.4	29.3			23.2				33.4	38.0	27.9							
>18– ≤30g/d	13.2	5.8	13.0	14.5			14.3				14.7	14.2	11.5							
>30g/d	19.2	7.6	12.5	15.4			13.8				12.8	27.6	11.9							
Dietary factors (g/d)																				
Fruits and vegetable	375 (204)	481 (227)	320 (144)	311 (147)		510 (229)					290 (135)	364 (200)	525 (289)							
Red and processed meat	95 (52)	66 (42)	103 (48)	95 (54)		79 (42)					98 (57)	104 (51)	74 (49)							
Poultry	20 (18)	27 (20)	10 (9)	12 (11)		27 (20)					12 (11)	22 (18)	21 (21)							
Fish	32 (25)	36 (26)	10 (9)	11 (11)		30 (21)					22 (23)	42 (24)	36 (32)							
Milk and yogurt	291 (261)	388 (179)	349 (264)	287 (280)		189 (173)					181 (207)	326 (291)	238 (204)							
Pasta and rice	56 (65)	44 (43)	54 (44)	62 (56)		168 (112)					18 (15)	50 (38)	69 (55)							
Bread	147 (76)	86 (58)	153 (65)	156 (75)		160 (91)					178 (79)	147 (66)	124 (77)							
Vegetable oil	6.6 (10.7)	4.4 (3.2)	3.0 (3.3)	4.5 (4.0)		31.3 (13.8)					4.2 (3.4)	2.7 (3.6)	13.1 (16.4)							
Butter and margarine	20.3 (16.0)	20.6 (16.4)	25.3 (15.5)	22.6 (15.9)		2.3 (3.9)					26.8 (16.5)	20.8 (14.5)	11.9 (14.5)							
Chocolate	9.0 (13)	13.1 (16.6)	9.6 (11.5)	10.5 (12.5)		4.3 (8.1)					12.4 (15.5)	8.0 (11.9)	8.1 (15.1)							
Cake and cookies	38 (44)	67 (62)	30 (22)	28 (23)		52 (49)					61 (57)	20 (20)	43 (43)							
Soft drinks	101 (191)	120 (190)	105 (124)	122 (145)		23 (70)					46 (136)	131 (229)	44 (119)							

Data are means (SD) or percentages. Bl  =  Baseline.Table 3. Combined estimates of relative risk for the association of retained predictors with substantial weight gain*.

The pooled estimates of relative risk for the association of included predictors with risk of SWG and corresponding score points assigned to each predictor are presented in **Table 2**. The pooled estimate of the background probability of avoiding SWG (analogous to ‘survival’) at 5 years estimated at average values of the predictors was 0.9331, implying that under average conditions about 93% of the population will stay free of SWG while 7% will experience SWG within 5 years. For each participant, the probability of SWG during the next 5 years [P(SWG,5y)] was calculated by inserting the individual's risk score into the following survival function while correcting for the averages of the participants’ risk factors:
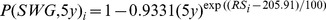



**Table 2:** Combined estimates of relative risk for the association of retained predictors with substantial weight gain.*

Predictor	β	Hazard Ratio (95% CI) *	Points allocated
Age (per year)	−0.03498	0.97 (0.96–0.97)	−3.50
Sex (female vs. male)	0.26477	1.30 (1.02–1.66)	26.48
Baseline weight (per kg)	−0.01719	0.98 (0.98–0.99)	−1.72
Technical school (vs. none)	−0.14118	0.87 (0.81–0.93)	−14.12
Secondary school (vs. none)	−0.13418	0.87 (0.76–1.004)	−13.42
University (vs. none)	−0.25475	0.78 (0.70–0.86)	−25.48
Current smoking (vs. current non-smoking)	0.39101	1.48 (1.32–1.65)	39.10
Sports (per h/week)	−0.03939	0.96 (0.94–0.98)	−3.94
No alcohol (vs. >0−<6g/d)	0.12682	1.14 (1.01–1.28)	12.68
Alcohol >6 to ≤18g/d (vs. >0–<6g/d)	−0.20401	0.82 (0.74–0.90)	−20.40
Alcohol >18 to ≤30g/d (vs. >0–<6g/d)	−0.23064	0.79 (0.66–0.95)	−23.06
Alcohol >30g/d (vs. >0−<6g/d)	−0.21749	0.80 (0.67–0.96)	−21.75
Red and processed meat (per 100g/d)	0.14967	1.16 (1.09–1.24)	14.97
Poultry (per 50g/d)	0.13675	1.15 (1.05–1.25)	13.68
Fish 100g/d)	0.16171	1.18 (0.996–1.39)	16.17
Bread (per 50g/d)	−0.03779	0.96 (0.94–0.99)	−3.78
Cake and biscuits (per 50g/d)	−0.09724	0.91 (0.84–0.98)	−9.72
Soft drinks (per 250g/d)	0.08404	1.09 (1.03–1.14)	8.40

* Predictors were identified using center-specific stepwise Cox regression in the derivation sample. Those factors being significantly (two-sided P-value <0.05) related to substantial weight gain in ≥2 centers were retained for the final model. Center-specific effects for the retained predictors were pooled using random-effects meta-analysis. These combined estimates of relative risk are presented in the table. For continuous variables, relative risks per increase of a defined portion size are presented. For categorical variables, comparison with the reference group is shown. Substantial weight gain was defined as gaining ≥10% of baseline weight during the individual's follow-up.

The probability of experiencing SWG within the following 5 years for 100, 150, 200, 250, 300, 350, and 400 score points was 2.4, 3.9, 6.3, 10.2, 16.3, 25.4, and 38.3%, respectively. The discriminatory ability of the model measured by the c index (95% CI) was 0.64 (0.63–0.65) in the derivation sample. This means that individuals who experienced SWG during 5 years had higher predicted risks than persons not experiencing SWG in 64% of the cases. The discriminatory accuracy showed some variation across centers, with c indexes (95% CI) ranging from 0.64 (0.62–0.65) in DK-Copenhagen/Aarhus to 0.71 (0.68-0.75) in NL-Amsterdam/Maastricht. The overall discriminatory accuracy in the validation sample was 0.57 (0.56–0.58). Similarly to the observation for development sample, it differed across single centers, varying between 0.56 (0.55–0.57) in France and 0.67 (0.64–0.71) in IT-Naples. In addition to between-center differences, the score generally performed better among men than women (**Table 3**), while the additional inclusion of menopausal status at recruitment did not affect the observed discriminatory accuracy in women across centers (data not shown).

**Table 3:** Sensitivity, specificity, positive and negative predictive value for various cut-off points of the risk score in the derivation sample.

Score points	Percentage of the population	Sensitivity (%)	Specificity (%)	Youden's index (J)	PPV (%)	NPV (%)
≥100	99.5	100.0	0.8	0.035	6.7	99.8
≥125	96.3	99.4	4.1	0.090	6.9	99.0
≥150	87.8	96.0	13.0	0.150	7.3	97.8
≥175	74.2	87.9	27.0	0.199	7.9	96.9
≥200	55.1	73.5	46.4	0.208	8.9	96.1
≥225	34.2	52.2	67.3	0.195	10.2	95.2
≥250	17.8	31.2	83.3	0.145	11.7	94.4
≥275	7.4	15.2	93.3	0.085	13.9	93.9
≥300	2.4	6.0	97.8	0.038	16.4	93.6
≥325	0.5	1.7	99.6	0.013	13.6	93.4

PPV  =  positive predictive value; NPV  =  negative predictive value. Youden's index was calculated according to the following formula: J  =  (sensitivity (%) + specificity (%) –100)/100.

Information on sensitivity, specificity and predictive values according to various cut-off points of the score in the derivation sample suggested a threshold of ≥200 points as the optimal cut-off value to define high-risk individuals (Youden's index, J = 0.208) (**Table 3**). This threshold captured 74% of the cases who experienced SWG. Furthermore, 46% of the persons who did not experience SWG had a score <200. The corresponding positive and negative predictive values were 9% and 96%, respectively.

**Figure 2:**
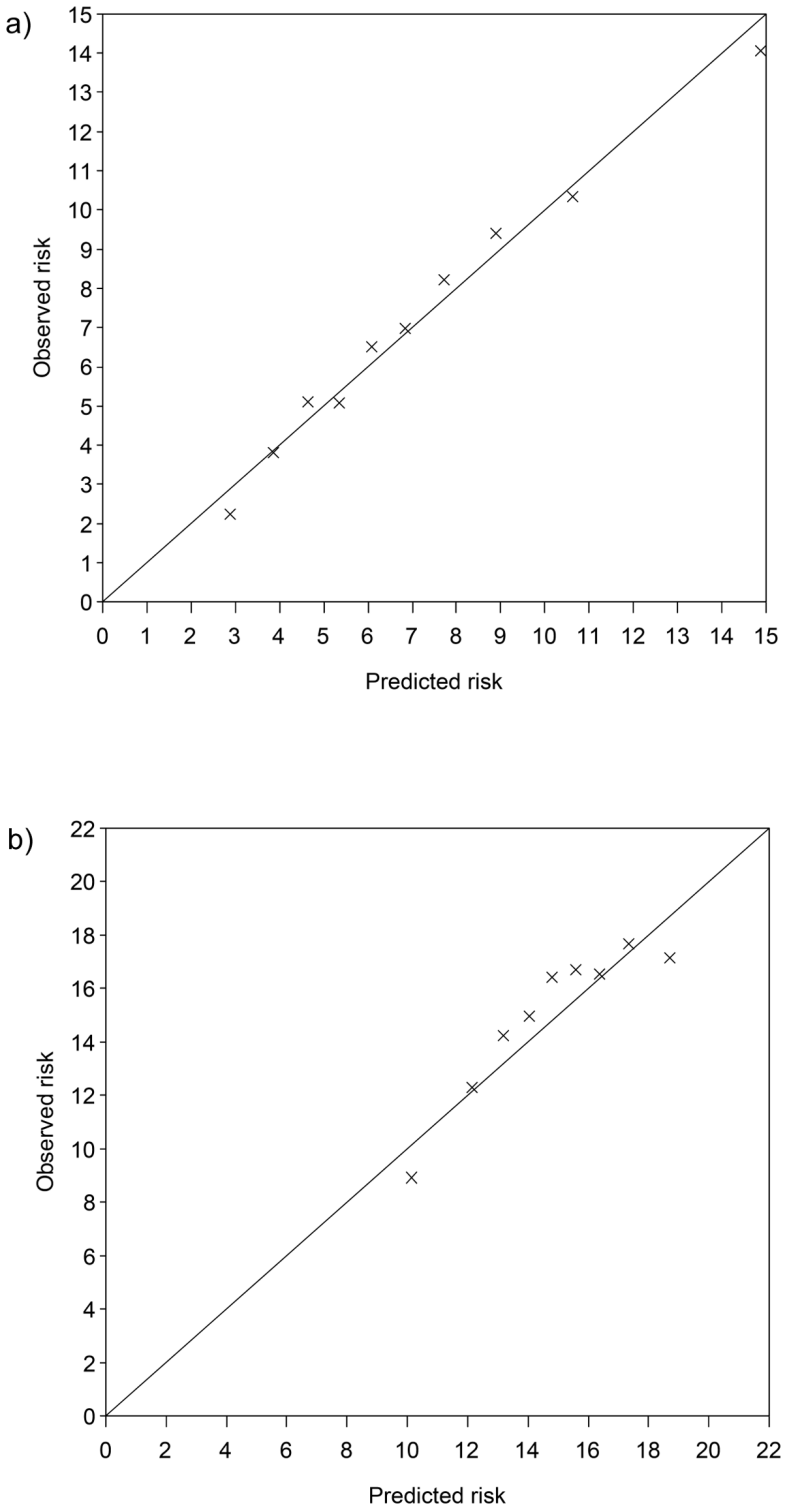
Calibration plot showing observed proportion of cases across tenths of predicted risk in the a) derivation sample and b) validation sample. Corresponding range of points for tenths in the derivation sample were <145, 145–<165, 165–<181, 181–<194, 194–<206, 206–<218, 218–<231, 231–<246, 246–<267, and ≥267. P for calibration  = 0.02. Corresponding range of points for tenths in the validation sample <162, 162–<185, 185–<200, 200–<212, 212–<223, 223–<234, 234–<246, 246–<259, 259–<280, and ≥280. P for calibration  = <001.

The estimated probability of experiencing SWG during 5 years agreed very well with the observed proportion of incident cases across tenths of predicted risk in the derivation sample although there was a slight overestimation of risk in the highest and lowest tenths of risk (**Figure 2a, p** **= 0.02**). In the total validation population, the score was also able to adequately quantify absolute risk, though comparison of observed and predicted risk implied a slight overestimation of risk in the lower and upper range of the score values and a slight underestimation in the middle range of the score (**Figure 2b, p** **<.001**). Inspection of calibration plots for each validation center indicated good calibration for the centers of Greece, UK-Health Conscious, UK-General Population and NL-Utrecht and adequate calibration in France and SWE-Malmoe (data not shown). In GER-Heidelberg we observed a systematic overestimation of risk, while in Spain calibration was poor, but no clear pattern of miscalibration was found.

The use of center–specific weights led to a marked improvement in discriminatory accuracy in the validation centers of France, Spain, Greece and GER-Heidelberg (**Table 4** **)**. When center-specific risk scores were developed (based on center-specific selection strategy), model performance remained essentially unchanged in comparison to the re-estimated model for all centers. The only exception was France for which discrimination improved from 0.61 (0.60–0.62) to 0.65 (0.63–0.67). Calibration generally improved or remained unchanged in the re-estimated model across validation centers (data not shown). Exceptions were the centers of GER-Heidelberg where risk remained continuously overestimated and Spain showing over- and underestimations of risk. Even in center-specific models, agreement between observed and predicted risk did not improve for those two centers.

**Table 4:** Discriminatory ability of the overall risk score across centers compared to the re-estimated overall model and center-specific models in the derivation and validation sample.

	Overall model	Re-estimated overall model *	Center-specific prediction models †
	aROC (95% CI)	aROC (95% CI)	aROC (95% CI)
**Derivation sample**			
**Total**	0.64 (0.63–0.65)	–	–
**UK-Norfolk**	0.66 (0.63–0.68)	0.67 (0.65–0.70)	0.68 (0.66–0.70)
**NL-Doetinchem**	0.65 (0.62–0.68)	0.67 (0.64–0.70)	0.66 (0.63–0.69)
**NL-Amsterdam/Maastricht**	0.71 (0.68–0.75)	0.66 (0.62–0.70)	0.71 (0.67–0.74)
**IT-Florence**	0.65 (0.62–0.68)	0.59 (0.55–0.62)	0.67 (0.64–0.70)
**GER-Potsdam**	0.67 (0.65–0.69)	0.62 (0.60–0.64)	0.67 (0.65–0.69)
**DK-Copenhagen/Aarhus**	0.64 (0.62–0.65)	0.52 (0.51–0.54)	0.66 (0.64–0.67)
**Validation Sample**			
**Total**	0.57 (0.56–0.58)	–	–
**France**	0.56 (0.55–0.57)	0.61 (0.60–0.62)	0.65 (0.63–0.67)
**IT-Naples**	0.67 (0.64–0.71)	0.67 (0.64–0.71)	0.67 (0.63–0.70)
**Spain**	0.60 (0.59–0.62)	0.64 (0.62–0.65)	0.64 (0.63–0.66)
**UK-GP**	0.63 (0.60–0.66)	0.65 (0.62–0.68)	0.64 (0.61–0.67)
**UK-HC**	0.58 (0.57–0.59)	0.60 (0.59–0.61)	0.61 (0.60–0.62)
**NL-Utrecht**	0.61 (0.60–0.63)	0.62 (0.61–0.64)	0.62 (0.60–0.64)
**Greece**	0.60 (0.58–0.62)	0.65 (0.63–0.67)	0.65 (0.63–0.67)
**GER-Heidelberg**	0.66 (0.64–0.69)	0.70 (0.67–0.72)	0.70 (0.68–0.73)
**SWE-Malmö**	0.63 (0.62–0.65)	0.65 (0.63–0.66)	0.65 (0.63–0.66)

* Using center-specific regression coefficients for all predictor variables included in the overall model.

† Including only center-specific predictor variables.

## Discussion

In this large multi-center prospective study of middle-aged European men and women, a risk score based on numerous easily assessable socio-demographic, dietary and lifestyle factors was found to exhibit moderate discriminatory accuracy and ability to accurately predict risk of experiencing SWG during the following 5 years.

Major strengths of the present study are its prospective design, its large sample size, the availability of information on a large number of risk factors for weight gain, the use of multiple imputation techniques to avoid potential bias in derivation of the score and the validation of the risk score in several independent, culturally diverse study populations.

Some methodological limitations need to be considered. At follow-up, most participants provided self-measured weight. However, we tried to correct for potential underreporting by applying prediction equations [21]. Further, only two measurements of body weight were available for each individual and weight gain was considered linear, which is a strong assumption about the course of weight gain. Weight gain is reversible, and it is well known that body weight tends to fluctuate over time [38], which may lead to repeated cycles of weight loss and recovery [39], [40] that are not reflected in a two-point-in-time measurement. Fluctuations or non-linear weight gain in general may have resulted in misclassification of cases and non-cases and additionally in misspecification of the cases' time to event, which might have limited the performance of the obtained risk score model. Nevertheless, recent findings from the EPIC-Potsdam study based on 5 measurements of weight suggest that weight gain can be reasonably well approximated by a straight line over a follow-up period of 8 years on the population-level [41].

Directions of associations with SWG for some predictors in our model may be difficult to explain on a causal basis. It has to be kept in mind though that, in contrast to etiological studies trying to explain the cause of a disease, a prediction model aims to develop a good predictor to enable accurate predictions of the outcome [42]. Thus, predictors in a prediction model do not necessarily need to be well-established etiological factors with a strong biological background. They could also be a marker of other lifestyle factors which influence mechanisms that are implicated in the regulation of body weight. Thus, caution may be warranted to avoid misinterpretation of the identified predictors in terms of driving weight gain. Regarding the positive association of baseline smoking with SWG, for example, we explored in a sub-analysis that this relation was driven by the strong weight-increasing effect of smoking cessation during follow-up, while continuous smoking was not related to a higher risk of SWG compared to non-smoking. This finding may be kept in mind when interpreting the results and emphasizes that weight management is warranted among individuals who attempt to quit smoking. Nevertheless, because future changes in smoking habits are unknown at the time of prediction, only baseline variables were included in the prediction model.

The discriminatory ability of the score was generally low to modest which may be explained by lack of information on some predictors in this analysis. Specifically, weight loss attempts [43], weight cycling [44], [45] and large short-term weight changes [38], [45] have been shown to determine future weight change. However, to obtain this type of information, a closer contact between participants and study personnel is required and assessment of this information in all centers of such a large study is challenging. Also, despite recent weight history may predict weight change in the near future, it is currently unknown whether this information is a strong factor to predict weight change over longer periods, e.g. 5 years.

In the field of chronic diseases, hopes have been raised that information on common genetic markers may be used to improve discriminatory accuracy beyond non-invasive factors and biochemical measures [11], [46]. The predictive ability of genetic factors, however, currently appears limited [11], [46]. For example, the addition of seven SNP's to the breast cancer model developed by Gail et al. only modestly improved discriminatory accuracy [46]. Similarly, the additional inclusion of 20 diabetogenic SNP's did barely improve discrimination of incident type 2 diabetes beyond lifestyle factors and metabolic markers in the EPIC-Potsdam cohort [47]. In respect to obesity, the EPIC-Norfolk study reported that 12 obesity-susceptible loci explained 0.9% of variation in BMI, with a c index of 0.57 for prediction of obesity [48]. Thus, despite overwhelming statistical significances and repeated replications, the explained variance and the predictive value of the currently identified obesity-susceptibility loci is low [49] and a considerable improvement of the model's accuracy due to inclusion of genetic markers appears unlikely. Additionally, it should be noted that very large independent relative risks are needed for a single predictor to meaningfully improve discrimination [46].

The discriminatory ability of the present risk score was reduced in the external validation sample, an observation that is also commonly reported for external validation studies in the field of chronic diseases [10], [11]. Several reasons may be thought of to explain this phenomenon. First, overfitting of the model in the derivation sample may be responsible for the poorer performance in the validation sample; however, given that the sample size of the development sample was large and that the amount of optimism decreases with larger sample size [50], this explanation appears unlikely. Second, lower predictive accuracy in external populations may be due to differences between the derivation and validation population, especially with regard to methods of data collection, coding of predictors and endpoint, and the availability of all variables used to construct the score [50]. However, given the standardised methodology followed in EPIC, this explanation also seems rather unlikely. To account for the fact that some validation centers were sampled from specific groups rather than the general population, e.g. France, IT-Naples, NL-Utrecht and UK-Oxford, which may affect the model's performance, we excluded those centers in sensitivity analyses. Nevertheless, the overall discriminatory accuracy did barely change (0.59 (0.58–0.60)). Interestingly, apart from the overall difference in predictive ability between derivation and validation sample, there was considerable variation in discrimination across single cohorts of the derivation and validation sample, respectively. Specifically, discriminatory power ranged from 0.64 in UK-Cop./Aarhus to 0.76 in NL-AmMa in the derivation set and varied between 0.56 (France) and 0.67 in IT-Naples in the validation set. It is further noteworthy that a comparable predictive accuracy was exhibited among centers of similar socio-cultural background in the derivation and validation sample (e.g. in Denmark and Sweden, in Potsdam and Heidelberg). This suggests the prediction of weight gain to depend on underlying socio-cultural factors that were not similarly represented by the predictors included in the present model across the trans-European study populations.

The risk score adequately estimated risk in the total validation sample, while in some of the centers calibration was poor. It has been suggested that adjusting or re-calibration of the score to the local circumstances in external populations may increase the predictive performance. In the present study, re-estimation of regression coefficients slightly improved calibration in most validation centers except for GER-Heidelberg and Spain in which calibration remained poor even in center-specific models. An explanation for this finding may be the considerably shorter follow-up time in the two centers. While our prediction model was tailored to the time period of 5 years, average observed follow-up times in GER-Heidelberg and Spain were 2.1 and 3.3 years, respectively. Unfortunately, we did not have access to more recent data to further investigate this issue. Discriminatory ability markedly improved in four of the nine validation centers in the re-estimated model, whereas center-specific models did generally not lead to further improvements in discriminatory ability. The only exception was the center of France for which a population-specific model yielded a c index of 0.65 (0.63–0.67) compared to 0.61 (0.60–0.62) in the re-estimated model and 0.56 (0.55–0.57) in the overall model.

Despite the observed improvements in discrimination using re-estimation of parameters, the performance measures were generally moderate. Although we cannot rule out the possibility that important, maybe population-specific, predictors may not have been assessed in this study, our findings based on a wide range of predictors and several culturally diverse study populations rather convey the impression that the predictability of weight gain based on data from large population-based studies might be limited in general.

Test characteristics of the risk score also challenge its practical implementation into prevention programs. The optimal cut-off value to define high-risk individuals was ≥200 points and implies that preventive actions will be indicated for a substantial part of the population (55%). Of these high-risk individuals, 9% will indeed experience SWG within 5 years. On the other hand, 96% of the individuals with a score <200 will indeed not develop SWG. It is of note that the optimal cut-off point was exemplarily defined using the Youden's index and for its calculation, sensitivity and specificity are considered as equally important. This however might not hold true in practice. When implementing a risk score in practice, designation of a cut-off value should depend on the importance attached to false-positives and false-negatives accounting for misclassification costs.

In conclusion, the present risk score was able to confidently exclude a large proportion of individuals from being at any appreciable risk to develop SWG within the next 5 years. Future studies, however, may attempt to further refine the positive prediction of the score by for example considering additional predictors both in general and on the national level.
